# Attitudes of Postgraduate Students Towards Distance Education During the COVID-19 Pandemic: North Cyprus Example

**DOI:** 10.3389/fpsyg.2021.766183

**Published:** 2021-10-18

**Authors:** Yeşim Üstün Aksoy

**Affiliations:** Department of Educational Administration and Supervision, Near East University, Nicosia, Cyprus

**Keywords:** COVID-19, education during the pandemic, online education, higher education, postgraduate studies

## Abstract

The distance education model has become the most common educational model used around the world due to the COVID-19 pandemic that emerged in China at the end of 2019. In North Cyprus (NC), traditional face-to-face education had been resumed through online channels. The rapid shift to online channels has not only caused distress among students who do not have experience with using in online education systems, but also caused many problems to surface in terms of access to education. This study aims to explore the attitudes and views of higher education students in NC regarding the distance education implemented during the COVID-19 pandemic in the 2020–2021 educational year. In this quantitative study, the survey method was used. A random sampling approach was used for determining the study group, which was formed of 470 volunteer higher education students. According to the analysis of the data collected from the students, it was determined that they received an average score of *x̅*=82.52±19.50 points from the overall Attitudes Regarding the Use of Distance Education Environments During the Pandemic (ASRUDEEDP) scale, *x̅*=23.03±7.21 from the competence and education sub-dimension, *x̅*=26.36±5.81 from the practicality sub-dimension, *x̅*=18.20±4.83 points from the efficiency sub-dimension, and *x̅*=14.93±4.12 from the satisfaction sub-dimension. It was identified that there were no differences in the scores according to the age group, grade, or Internet use durations (*p*>0.05) but the scores of male students were observed to be higher than those of female in the sub-dimensions of competence and motivation as well as practicality within the ASRUDEEDP.

## Introduction

COVID-19 disease is caused by the novel coronavirus (SARS-CoV-2) that emerged in Wuhan, China, at the end of December 2019. Carrying high risks of infection, the virus has spread across the whole world, primarily to Europe [World Health Organization (WHO), 2020]. During this process, recommendations to close educational institutions to minimize the high risk of spread among the communities were taken into consideration (Kawano and Kakehashi, 2015; De Luca et al., 2018). Within this context, the decision was made to temporarily shut down schools, universities, and other educational institutions in many countries to slow down the rate at which the COVID-19 pandemic was spreading. After the Health Ministry announced the first incidence of COVID-19 in the TRNC on March 11, 2020, the schools and educational institutions were temporarily closed on March 25 (TRNC Health Ministry, 2020).

For the management of this process and the crisis, the Higher Education Institution (YÖK) swiftly made the decision to transform the conventional education to distance education for the upcoming spring semester of the 2020 education year. As a result of this announcement, all levels of conventional education were halted; thus, distance education began to be implemented along with cancelation of local and central examinations, which were later replaced by web-based examinations for the assessment of the level of student efficiency. Within this context, the system that had been constructed based on conventional education had to be transformed into web-based distance education as a result of successful crisis management.

The developments of our era, particularly in the areas of technology and science, enhance the tendency for adaptation to the era and the desire for education. However, educational systems have resisted the need to adapt to the innovations in the areas of technology and science and have therefore been criticized for not being sufficiently innovative (Akdemir et al., 2020). Being a society that produces and evolves in the information era in which we are living is only possible by becoming an information society. Being an information society, on the other hand, is only possible with individuals who continuously reach and develop through information. This development within individuals is possible through lifelong education. However, it is not possible for full-time working adults to access education opportunities that could facilitate their personal development in a conventional education model.

Distance education is a discipline aimed at eliminating the limitations of the sources for teachers, students, and education while using current technologies (Bozkurt, 2017). In general, distance education can be defined as a dedicated, complex, hierarchic, and unilinear education system.

When the definitions of distance education are taken into consideration, the California Distance Learning Project (CDLP) defines the distance education programs as system that actualize education by making connections between the student and educational resources, whereas the United States Distance Learning Association (USDLA) defines it as “Fetching the education to students in different regions through satellite, videos, graphics, computers and multimedia-assisted electronical equipment” (Dinçer, 2017).

Distance Education is an education technology system implemented when the teacher and the student are not in the same place. According to a broader definition, it is an education type that provides connections through electronic communication media or printed materials when teachers and students are in different places (Aslantaş, 2011).

When the common features of the definitions are taken into consideration, it can be seen that distance education is a system that eliminates the notions of place and time between the teacher and student while benefiting from the advantages of technology through an effective way of accessing the information sources and facilitating to students. When the historical instances of the distance education are taken into consideration, it is found that applications date back to the 1700s. It is known that the first implementation of education through letters took place in Sweden in 1728. Also, according to information printed in a newspaper announcement in Sweden in 1833, education was planned to be delivered through letters (Çoban, 2012).

Today, associate degree, graduate, and postgraduate programs in many education facilities are conducted through distance education. Along with this, many universities apply a mixed education model through the use of both face-to-face and distance education methods. For many years, distance education applications have been carried out as “open education,” especially in the Anadolu University Open Plan Faculty. According to the Rules and Principles of Distance Education in Postgraduate Institutions published by YÖK, distance education applications have become more popular in universities (Cabı and Ersoy, 2017).

## Materials and Methods

### Model of the Research

Survey method is used in this quantitative study. This research is a supplementary study including scanning through survey method. According to Karasar (2015), survey models are research approaches which aim to depict a situation that once existed or currently exists as it is. The event, individual, or object which is the subject of the study is aimed to be depicted as it is under its very circumstances.

### Population and Sample

The population of this study was defined as postgraduate students in the Lefkoşa, Gazimağusa, Girne, and Lefke districts of North Cyprus. Convenience sample, as one of the sample types of quantitative research, is taken as a basis; thus, 470 students are reached in total.

In this study, 13.83% of the participant students were aged between 18 and 19, while 41.70% were between 20 and 21, 31.91% were between 22 and 23, and 12.55% were 24 or older; hence, the average age was 21.62±2.67, where 64.47% were female. It was determined that 25.96% of the students were in first grade, 22.77% were in second grade, 30.21% were in third grade, and 21.06% were in fourth grade. Additionally, 20.43% of the students stated that they used the Internet daily for 4h, whereas 26.60% use the Internet for 5 to 6h, 21.06% for 7 to 8h, and 31.91% for 8h or more.

### Data Collecting Tool

The attitudes regarding the use of distance education environments during the pandemic scale were used as the data collection tool. This scale was developed by Yıldız et al. (2021). It is composed of a total of 24 items and is a five-point Likert-type scale. The scale has a five-point structure type, namely, I strongly agree=5, I agree=4, I am neutral=3, I disagree=2, and I strongly disagree=1. Exploratory factor analysis (EFA) was conducted in order to determine the distribution of the scale items on the study group. According to the results of the EFA, the number of items in the scale was reduced from 25 to 24, with the eigenvalue greater than 1, considering that it disrupted its four-factor structure. The four-factor structure of the scale, consisting of 24 items with an eigenvalue greater than 1, indicates 73.42% of the total variance. A variance rate greater than 30% is considered sufficient for test developing studies in behavioral sciences (Rennie, 2012; Büyüköztürk, 2018). The results obtained from the validity and reliability analyses demonstrate that the scale has a consistent structure within itself.

### Analysis of the Data

Statistical Package for Social Sciences (SPSS) 24.0 software was used in the statistical analysis of the research data. Cronbach’s alpha test, which is an internal consistency test, was applied within the scope of reliability study for the answers given by the participant students to ASRUDEEDP, and the alpha coefficient for the overall scale was determined as 0.941.

The socio-demographical distribution of the students was determined through frequency analysis, and descriptive statistics were given regarding the scores that the participant students obtained from the ASRUDEEDP.

In order to compare the scores of the students in ASRUDEEDP according to their socio-demographical features, the compliance of the scale scores with the normal distribution was firstly examined through the Kolmogorov–Smirnov test, Q-Q plot graph, and skewness-kurtosis values, and it was determined that they conformed to normal distribution. Therefore, parametric hypothesis tests were used in the study; for example, the independent sample *t* test was used when the independent variable consisted of two categories, and ANOVA was used when it consisted of three categories or more.

### Findings

The descriptive statistics of the socio-demographical features of the participant students are listed below as well as the effects of their ages, sexes, grades, and Internet use durations on ASRUDEEDP and *t* test and ANOVA results that would highlight the differences among them, if they exist.

The descriptive statistics of the scores of the students from ASRUDEEDP and the ANOVA results based on a comparison of the ASRUDEEDP scores according to their age groups are shown in Table 1.

**Table 1:** Comparison of the students’ scores from ASRUDEEDP according to their age group.

	Age group	*N*	*x̅*	*S*	Min	Max	*F*	*p*
Competence and Motivation	18–19years	65	21.52	6.96	7	35	2.401	0.067
	20–21years	196	22.62	7.04	7	35		
	22–23years	150	23.62	7.15	7	35		
	24years and older	59	24.56	7.94	7	35		
Practicality	Total 18–19years	470 65	23.03 26.45	7.81 6.20	7 8	35 39	1.514	0.210
	20–21years	196	25.76	5.62	8	40		
	22–23years	150	26.70	5.87	8	40		
	24years and older	59	27.41	5.78	14	40		
Efficiency	Total 18–19years	470 65	26.36 17.38	5.81 4.93	8 5	40 25	0.903	0.439
	20–21years	196	18.14	4.76	5	25		
	22–23years	150	18.49	4.68	5	25		
	24years and older	59	18.53	5.27	5	25		
Satisfaction	Total 18–19years	470 65	18.20 14.65	4.83 4.46	5 4	25 20	0.336	0.799
	20–21years	196	14.86	3.92	4	20		
	22–23years	150	14.98	4.13	4	20		
	24years and older	59	15.36	4.43	4	20		
ASRUDEEDP	Total 18–19years	470 65	14.93 80.00	4.12 20.11	4 24	20 112	1.376	0.249
	20–21years	196	81.38	18.94	26	120		
	22–23years	150	83.79	19.30	24	120		
	24years and older Total	59 470	85.85 82.52	20.94 19.50	31 24	120 120		

Considering the results shown in Table 1 in the total points, it is determined that students received an average score of *x̅*=82.52±19.50 from the overall ASRUDEEDP, *x̅*=23.03±7.21 from the competence and motivation sub-dimension, *x̅*=26.36±5.81 points from the practicality sub-dimension, *x̅*=18.20±4.83 from the efficiency sub-dimension, and *x̅*=14.93±4.12 points from the satisfaction sub-dimension.

When Table 1 is evaluated, it is determined that there is no statistically significant difference between the overall ASRUDEEDP scores of the students and in the sub-dimensions of competence and motivations, practicality, efficiency, and satisfaction in the scale according to age group.

Table 2 shows the independent sample *t* test results for the comparison of the overall ASRUDEEDP score according to gender.

**Table 2:** Comparison of the students’ scores from ASRUDEEDP according to their gender.

	Gender	*N*	*x̅*	*s*	*t*	*p*
Competence and motivation	Female	303	22.54	6.99	−1.984	0.048*
	Male	167	23.92	7.54		
Practicality	Female	303	25.85	5.45	−2.595	0.010*
	Male	167	27.29	6.33		
Efficiency	Female	303	18.17	4.83	−0.186	0.853
	Male	167	18.25	4.83		
Satisfaction	Female	303	15.08	4.11	1.067	0.286
	Male	167	14.66	4.14		
ASRUDEEDP	Female	303	81.64	18.88	−1.322	0.187
	Male	167	84.12	20.53		

* *p*<0.05.

According to the results in Table 2, it is determined that there is statistically significant difference between the scores of the students in the competence and motivation sub-dimension and the practicality sub-dimension (*p*<0.05). Male students scores were higher than female students’ scores in the sub-dimension of competence and motivation throughout the ASRUDEEDP. There is also no significant difference between the overall ASRUDEEDP scores of the students and the sub-dimensions of the efficiency and satisfaction according to their gender (*p*>0.05).

Table 3 shows the ANOVA results of the comparison between the overall ASRUDEEDP scores of the students according to their grades.

**Table 3:** Comparison of the students’ scores from ASRUDEEDP according to their grades.

	Grade	*n*	*x̅*	*S*	Min	Max	*F*	*p*
Competence and Motivation	First grade	122	22.11	6.80	7	35	1.021	0.383
	Second grade	107	23.63	7.61	9	35		
	Third grade	142	23.39	7.03	7	35		
	Fourth grade	99	22.99	7.51	7	35		
Practicality	First grade	122	26.39	5.55	8	40	0.964	0.409
	Second grade	107	25.76	5.41	8	40		
	Third grade	142	26.26	6.33	8	40		
	Fourth grade	99	27.12	5.78	8	40		
Efficiency	First grade	122	17.77	4.65	5	25	0.838	0.473
	Second grade	107	18.54	4.86	5	25		
	Third grade	142	18.51	4.73	5	25		
	Fourth grade	99	17.89	5.14	5	25		
Satisfaction	First grade	122	14.91	4.10	4	20	0.658	0.578
	Second grade	107	15.38	4.07	4	20		
	Third grade	142	14.84	4.07	4	20		
	Fourth grade	99	14.61	4.29	4	20		
ASRUDEEDP	First grade	122	81.19	18.62	24	120	0.277	0.842
	Second grade	107	83.31	19.09	26	120		
	Third grade	142	83.01	19.92	25	120		
	Fourth grade	99	82.61	20.57	24	120		

When Table 3 is evaluated, it is determined that there is no significant difference between the overall ASRUDEEDP scores of the students according and the sub-dimensions of the scale according to their grades (*p*>0.05).

Table 4 indicates the ANOVA results of the comparison of the overall ASRUDEEDP scores of the students regarding their daily Internet use durations.

**Table 4:** Comparison of the students’ scores from ASRUDEEDP according to their daily Internet use durations.

	Daily Internet use duration	*N*	*x̅*	*s*	Min	Max	*F*	*p*
Competence and Motivation	4h or less	96	22.90	7.55	7	35	0.319	0.812
	5–6h	125	22.95	7.06	7	35		
	7–8h	99	23.65	6.68	7	35		
	8h and more	150	22.77	7.49	7	35		
Practicality	4h and less	96	25.30	5.64	8	38	1.488	0.217
	5–6h	125	26.38	5.23	8	40		
	7–8h	99	26.90	5.66	8	40		
	8h and more	150	26.67	6.42	8	40		
Efficiency	4h or less	96	17.93	5.07	5	25	0.159	0.924
	5–6h	125	18.15	5.06	5	25		
	7–8h	99	18.34	3.90	5	25		
	8h and more	150	18.31	5.05	5	25		
Satisfaction	4h or less	96	15.39	4.09	4	20	0.609	0.609
	5–6h	125	14.69	4.05	4	20		
	7–8h	99	15.02	3.71	4	20		
	8h and more	150	14.79	4.46	4	20		
ASRUDEEDP	4h or less	96	81.51	19.79	24	116	0.265	0.851
	5–6h	125	82.18	18.66	27	120		
	7–8h	99	83.91	17.76	24	120		
	8h and more	150	82.53	21.16	24	120		

When Table 4 is evaluated, it is indicated that there is no statistically significant difference between the overall ASRUDEEDP scores of the students and the sub-dimensions of competence and motivation, practicality, efficiency, and satisfaction points in the scale (*p*>0.05).

## Conclusion and Discussion

The rapid development of the technology, communication and transportation has accelerated globalization. In particular, the effects of the rapid spread of Internet technologies and communication are immense. Information is spread very quickly and shared through these channels. Naturally, these developments evolve according to the needs of all members of societies according to their age groups and information technologies impact all aspects of life. Due to the COVID-19 pandemic, the world has experienced a period in which it has been forced to adapt to new circumstances; therefore, the educational systems are being re-configured and online education is being integrated with face-to-face education. Online classes include providing the students with the leaning materials they needed which includes video tutorials, PowerPoint presentations, handouts etc. (Cortez, 2020). According to Doğan et al. (2017), the increase in the use of technology and the development of communication technologies facilitate the Internet access from all over the world along. From December 2019, parts of the world entered lockdown as a result of the COVID-19 pandemic, and the rapid spread of online education made immense contributions to the field of education (Talidong and Toquero, 2021). These new circumstances have increased the amount of time that students spend using the Internet. Considering the quantitative findings of the research, it is indicated that 80% of the university students use the Internet for 5h or more, 53% use the Internet for 7h or more, and 32% for 8h or more.

Even though the universities were largely unprepared to provide distance education during the COVID-19 pandemic, 49% of the students considered online education to be practical and sufficient, while 18% considered it to be effective and 15% were satisfied with this type of education. On the one hand, this demonstrates that particularly within the context of course explanation, distance education serves its purposes; however, in the researcher’s opinion, the lack of experience of teachers and students in this area might have decreased the efficiency in terms of pedagogical efficiency, and thus, the satisfaction rates of the students might have decreased either due to this or because of the lack of an environment that facilitates socialization. However, the general score totals obtained from the data indicate that the attitudes toward the use of distance education are high.

It was found that students’ attitudes toward the use of distance education environments in the pandemic generally increase with age; however, there is no statistically significant difference according to age groups. Considered separately in terms of competence and motivation, usefulness, efficiency, and satisfaction or as a whole, it is indicated that there is no statistically significant difference between age groups even though all university students’ attitudes toward educational mediums are higher when compared to competence and motivation, usefulness, efficiency, and satisfaction. When evaluated within the scope of daily Internet use durations, there is statistically significant difference among the attitudes. Similar to the age groups, no statistically significant difference is seen between the grades. This indicates that there is a parallelism between age and grades, which demonstrates consistency of the results and the credibility of the scale from another point of view.

Regarding the use of the distance education environments during the pandemic, in the practicality and competence and motivation sub-dimensions, the attitudes of the male students are statistically higher than the female students, and as this difference is statistically significant, this indicates that male students consider online education to be more practical and sufficient when compared to females and the reasons for that should be researched further. In the efficiency and satisfaction sub-dimensions, no statistically significant difference was found between the male and female students’ attitudes.

## Recommendations

The following recommendations can be listed according to the results of this study: Given that the effects of the COVID-19 pandemic will last for some time and the distance education will be implemented alongside face-to-face education, not only the educational programs but also the educational methods and techniques should be enhanced and enriched in the light of technological developments. In this context, proficiency techniques for evaluation should be reconsidered. Redesigning the pedagogical approaches and teaching programs taking online education into consideration along with the face-to-face education would beneficial in teacher trainings. In training new teachers in these new programs, on-the-job trainings should be provided, not only in postgraduate studies but also in lower education levels.

## Data Availability Statement

The original contributions presented in the study are included in the article/supplementary material, further inquiries can be directed to the corresponding author.

## Ethics Statement

The studies involving human participants were reviewed and approved by the Near East University Ethical Committee. The patients/participants provided their written informed consent to participate in this study.

## Author Contributions

YA wrote the manuscript and designed and completed the research report.

## Conflict of Interest

The author declares that the research was conducted in the absence of any commercial or financial relationships that could be construed as a potential conflict of interest.

## Publisher’s Note

All claims expressed in this article are solely those of the authors and do not necessarily represent those of their affiliated organizations, or those of the publisher, the editors and the reviewers. Any product that may be evaluated in this article, or claim that may be made by its manufacturer, is not guaranteed or endorsed by the publisher.
